# Platelet-rich plasma induces post-natal maturation of immature articular cartilage and correlates with LOXL1 activation

**DOI:** 10.1038/s41598-017-02297-9

**Published:** 2017-06-16

**Authors:** Yadan Zhang, Ben J. Morgan, Rachel Smith, Christopher R. Fellows, Catherine Thornton, Martyn Snow, Lewis W. Francis, Ilyas M. Khan

**Affiliations:** 1 Centre of NanoHealth, Swansea University Medical School, Swansea University, Singleton Park, Swansea, SA2 8PP United Kingdom; 2 Reproductive Biology Group, Swansea University Medical School, Swansea University, Singleton Park, Swansea, SA2 8PP United Kingdom; 3 Human Immunology Group, Swansea University Medical School, Swansea University, Singleton Park, Swansea, SA2 8PP United Kingdom; 4 School of Veterinary Medicine, Surrey University, Guildford, Surrey, GU2 7AL United Kingdom; 5 Royal Orthopaedic Hospital, Northfield, Birmingham B31 2AP United Kingdom

## Abstract

Platelet-rich plasma (PRP) is used to stimulate the repair of acute and chronic cartilage damage even though there is no definitive evidence of how this is achieved. Chondrocytes in injured and diseased situations frequently re-express phenotypic biomarkers of immature cartilage so tissue maturation is a potential pathway for restoration of normal structure and function. We used an *in vitro* model of growth factor-induced maturation to perform a comparative study in order to determine whether PRP can also induce this specific form of remodeling that is characterised by increased cellular proliferation and tissue stiffness. Gene expression patterns specific for maturation were mimicked in PRP treated cartilage, with chondromodulin, collagen types II/X downregulated, deiodinase II and netrin-1 upregulated. PRP increased cartilage surface cell density 1.5-fold (*P* < *0.05*), confirmed by bromodeoxyuridine incorporation and proportionate increases in proliferating cell nuclear antigen gene expression. Atomic force microscopy analysis of PRP and growth factor treated cartilage gave a 5-fold increase in stiffness correlating with a 10-fold upregulation of lysyl oxidase like-1 gene expression (*P* < *0.001*). These data show PRP induces key aspects of post-natal maturation in immature cartilage and provides the basis to evaluate a new biological rationale for its activity when used clinically to initiate joint repair.

## Introduction

Platelet-rich plasma (PRP) is increasingly used to treat acute and chronic musculoskeletal conditions such as osteoarthritis, though its mode of action and efficacy are subject to ongoing debate^1–3^. PRP is obtained from whole blood by serial centrifugation, first to separate plasma, then to enrich it for platelets five to eight-fold above baseline levels. When activated, alpha-granules within platelets release growth factors that influence the recruitment, migration, proliferation and differentiation of cells involved in tissue repair pathways^4^. In prospective clinical studies, improved outcomes over 12 months have been reported for patients suffering from chondral or osteoarthritic lesions following intra-articular injection of autologous PRP, but these improvements are generally not sustained long-term^5, 6^. More detailed analyses of PRP-treated osteoarthritic patients reveals a positive correlation between clinical outcome measures and, lower age, high activity and lower degree of cartilage loss^7, 8^, indicating a greater potential for healing in these particular cohorts. So far, only elementary cellular mechanisms have been proposed to explain how PRP might stimulate repair of injured or diseased cartilage; PRP promotes cellular proliferation, matrix synthesis, and modulates inflammatory signaling in cultured chondrocytes^9, 10^. Extrapolation of the latter mechanisms into the context of cartilage repair does not appear to explain either the poor efficacy of PRP generally, or, its positive effect in certain patient cohorts, suggesting its role in repair is more complicated than previously thought. Therefore, better understanding of the mechanisms that drive PRP-induced cartilage repair would allow refinement of PRP preparations, treatment regimes and patient selection to generate more effective and reproducible clinical outcomes.

In common with other tissues, cartilage repair and regeneration is thought to involve recapitulation of developmental morphogenesis^11, 12^. There are two major developmental epochs in generating adult cartilage, the first is embryonic joint formation that generates hyaline cartilage^13^, followed postpartum by a gradual maturation process that results in tissue uniquely adapted to its biomechanical environment^14^. Immature joint cartilage in newborns is composed of a relatively unstructured matrix within which chondrocytes appear to be randomly distributed^15^. As greater biomechanical stress is placed on joints during early growth, articular cartilage undergoes a radical change in architecture. Collagen, the major structural protein in cartilage is reconfigured to form arch-like fibrillar structures called Benninghoff’s arcades that form a stable, crosslinked framework within which a proteoglycan-rich extracellular matrix is embedded^15^. Chondrocytes align themselves along the collagen fibrils, typified by columnar chondrocytes in the deeper zones, to form a stratified structure that is stiffer and which is highly adapted to withstanding mechanical loading. Postnatal maturation of articular cartilage to attain the adult structural form takes approximately 10–18 years in humans^14^, and magnetic resonance imaging suggests following surgical repair of chondral lesions the same remodeling process takes at least two years^16^.

*In vitro* studies have shown that two growth factors, FGF2 and TGFβ1, initiate and accelerate the process of postnatal articular cartilage maturation approximately ten times the rate seen *in vivo*, so that immature bovine cartilage matures within 21 days^17^. Functional and biochemical analyses of *in vitro* growth factor-matured cartilage show it is indistinguishable from freshly isolated native adult cartilage^18^. Alpha-granules in platelets contain growth factors FGF2 and TGFβ1, and when activated have the potential to induce articular cartilage maturation by fusion with the platelet plasma membrane and release of their contents^19^. Numerous studies that have shown that chondrocytes in injured, repairing and diseased articular cartilages re-express, *in situ*, phenotypic biomarkers of immature tissue, and are therefore potentially receptive to maturation-related signals required to initiate reconstruction of adult-like cartilage^20–23^. In order to understand if development-related repair mechanisms are activated in the clinical setting, the first question we must answer is whether PRP can induce articular cartilage maturation and this was done by direct comparison with a previously validated *in vitro* model system^17^.

## Results

### PRP treatment induces chondrocyte proliferation in articular cartilage

**Figure 1:**
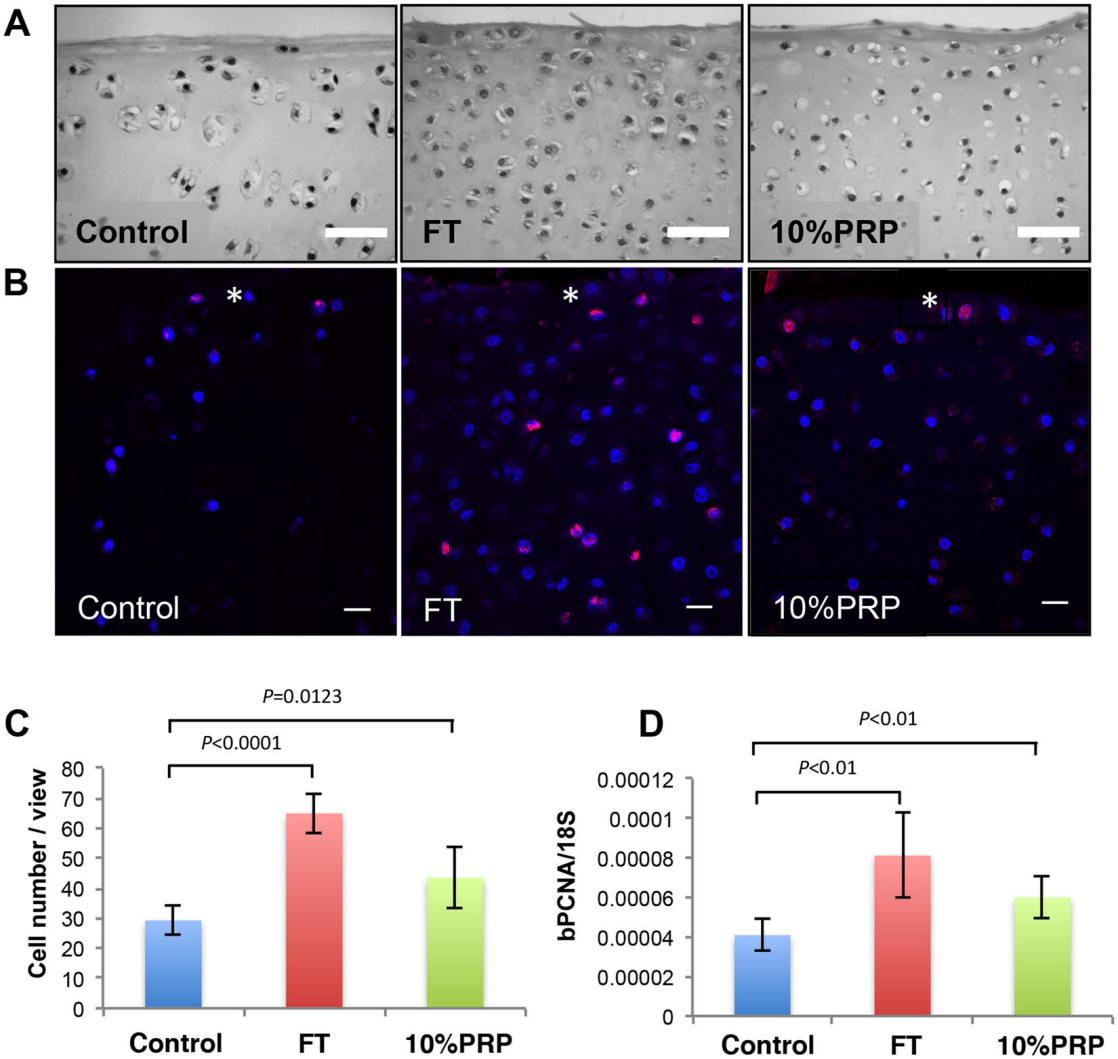
PRP and FGF2-TGFβ1 treatment of immature articular cartilage increases cell number in the surface zone of cultured bovine cartilage explants. 10% PRP lysate or growth factors FGF2 (100 ng ml^−1^) and TGFβ1 (10 ng ml^−1^) (FT) were added to serum-free culture medium with immature articular cartilage explants and cultured for 21 days. (**A**) Haemotoxylin and eosin staining of representative sections show the change in cell density at the surface of cartilage explants following treatment. Scale bar equals 50 μm. (**B**) Explants cultured for 21 days were exposed to 10 μM BrdU for 24 hours. BrdU-positive labelling was detected using a TRITC-conjugated secondary antibody (red) and nuclei counterstained using DAPI (blue). Cells positive for BrdU were detected as purple/pink labelled nuclei. The white asterisk marks the surface of the cartilage explant. Scale bar equals 20 μm. (**C**) Graph showing the cell number per microscopic field view (200 μm × 190 μm) for treated and untreated immature cartilage (n = 10 per group). (**D**) Graph of gene expression showing the absolute qPCR values for PCNA using 18 S rRNA as housekeeping control.

One of the pre-requisites for post-natal maturation is surface growth to generate neo-cartilage^14, 17^, so we measured the cellular density at the surface zone of cartilage explants cultured for 21 days in the presence of growth factors or 10% PRP lysate, Fig. 1A. FGF2-TGFβ1, 2.2-fold, and 10% PRP, 1.5-fold, significantly increased total cell number per microscopic field (FGF2-TGFb1, 65.0 ± 6.6, *P* < *0.0001* v control: 10% PRP, 43.7 ± 10.3, *P* = *0.0123* v control, n = 10), compared to untreated explants (29.3 ± 5.2, n = 10), Fig. 1C. We confirmed that the increase in cell number was due to cellular proliferation through the addition of the nucleotide analogue bromodeoxyuridine to explants where positively labeled nuclei were detected in the surface zones of all explants, Fig. 1B. Transcriptional analysis of proliferating cell nuclear antigen (PCNA) showed a proportionate 2-fold and 1.48-fold increase following treatment with growth factors FGF2-TGFβ1 and 10% PRP (*P* < 0.01), Fig. 1D. We also found that expression for ITGA3, a mesenchymal stem cell marker, was significantly increased following growth factor (28-fold) or 10% PRP treatment (54-fold) of cartilage explants (Supplemental Data 2). Antibody labelling for ITGA3 was localized to surface zone chondrocytes of PRP treated explants indicating an expansion in the number of fibronectin-binding cells compared to untreated cartilage.

### Maturation-specific gene expression patterns are mimicked following PRP treatment

**Figure 2:**
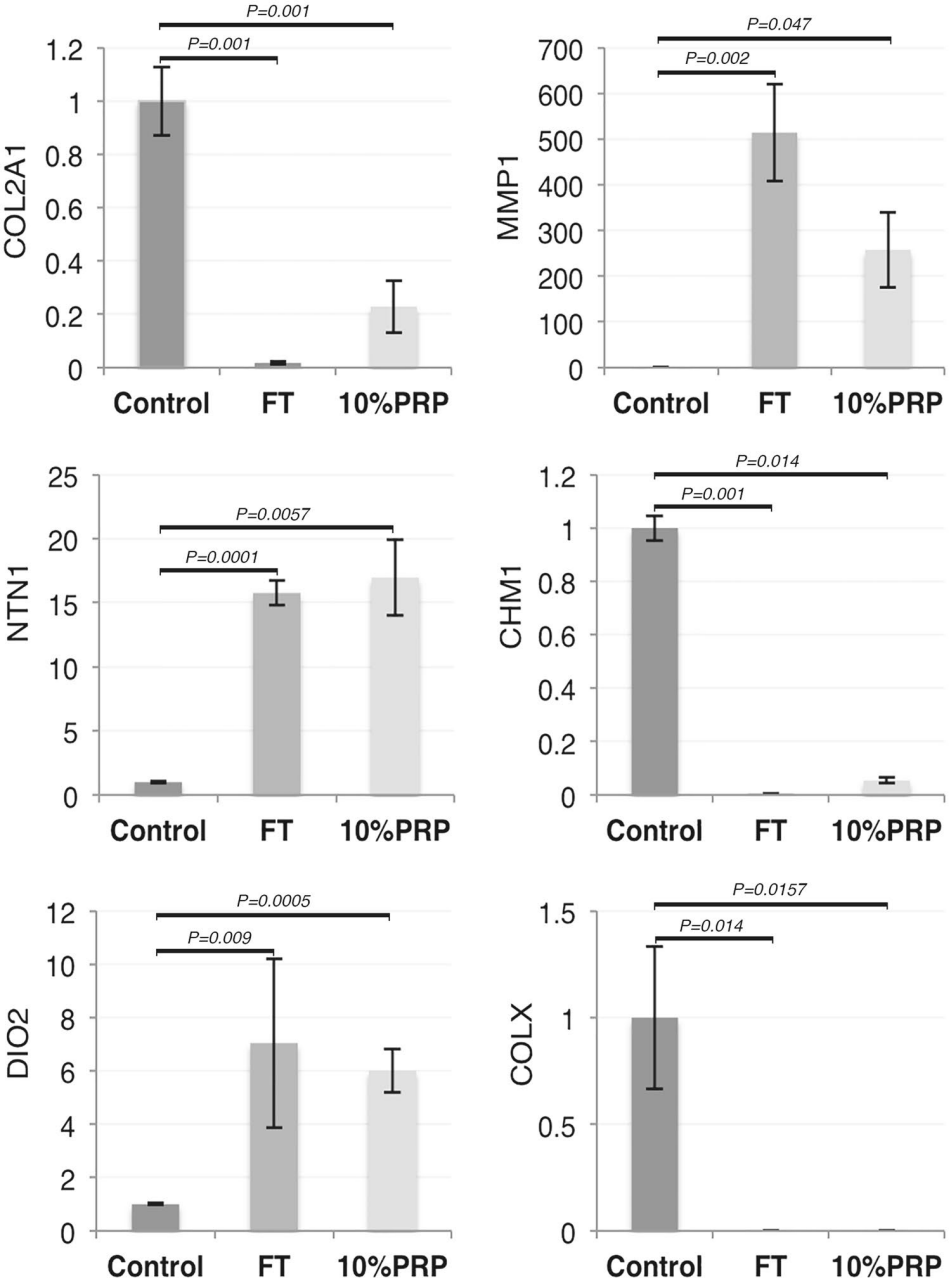
PRP lysate induces a maturation-like gene expression profile in immature articular cartilage. The relative fold change in gene expression of COL2A1, MMP1, NTN1, DIO2, CHM1 and COLX following growth factor or 10% PRP treatment are shown. Samples were tested after 14 days in culture (n = 4 for each group). Absolute quantitation (in ng) normalised to a 18S rRNA housekeeping gene was used to measure expression levels with the values converted to show relative-fold change in expression compared to untreated samples.

Immature cartilage cultured in PRP mimics the gene expression patterns that occur during growth factor-induced maturation. First we analysed the expression of genes that have been previously shown to be modulated during cartilage maturation^17^. Collagen type II (COL2A1) transcription was downregulated 57-fold in growth factor treated explants (*P* = *0.001*, n = 4) and 4-fold (*P* = *0.001*) in PRP treated explants, whilst matrix metalloproteinase-1 (MMP1) expression was upregulated 514-fold (*P* = *0.002*) after growth factor treatment and 257-fold (*P* = *0.05*) after culture with PRP, Fig. 2. Netrin-1 (NTN1), a gene involved in chondroprogenitor mobility^24^, increased approximately 13-fold in both growth factor (*P* < 0.0001) and 10% PRP treated explants (*P* = 0.0057). Similarly we also observed a 7-fold increase in deiododinase type II (DIO2) gene expression following growth factor-induced maturation (*P* = *0.0005*) and a 6-fold increase when explants were cultured in 10% PRP (*P* = *0.0091*). Deiodinases regulate the cellular activity of thyroxine, which has been shown to modulate cartilage maturation, in particular columnar chondrocyte formation^25^. Chondromodulin-1 (CHM1), a marker of immature cartilage^26^, expression fell 500-fold (*P* < *0.001*) following growth factor treatment and 10-fold when cultured in 10% PRP (*P* = *0.0142*), Fig. 2. Collagen type X (COL10), a marker of epiphyseal growth cartilage that is replaced by bone during maturation^27^, showed steeper declines in gene expression falling 1484-fold (*P* = *0.0141*) in growth factor and 25-fold (*P* = 0.0157) in 10% PRP treated cartilage explants. Only COL2 gene expression levels were significantly different between treated groups (*P* = *0.02*).

### Increased stiffness of PRP treated cartilage correlates with upregulation of LOXL1 gene and protein expression

**Figure 3:**
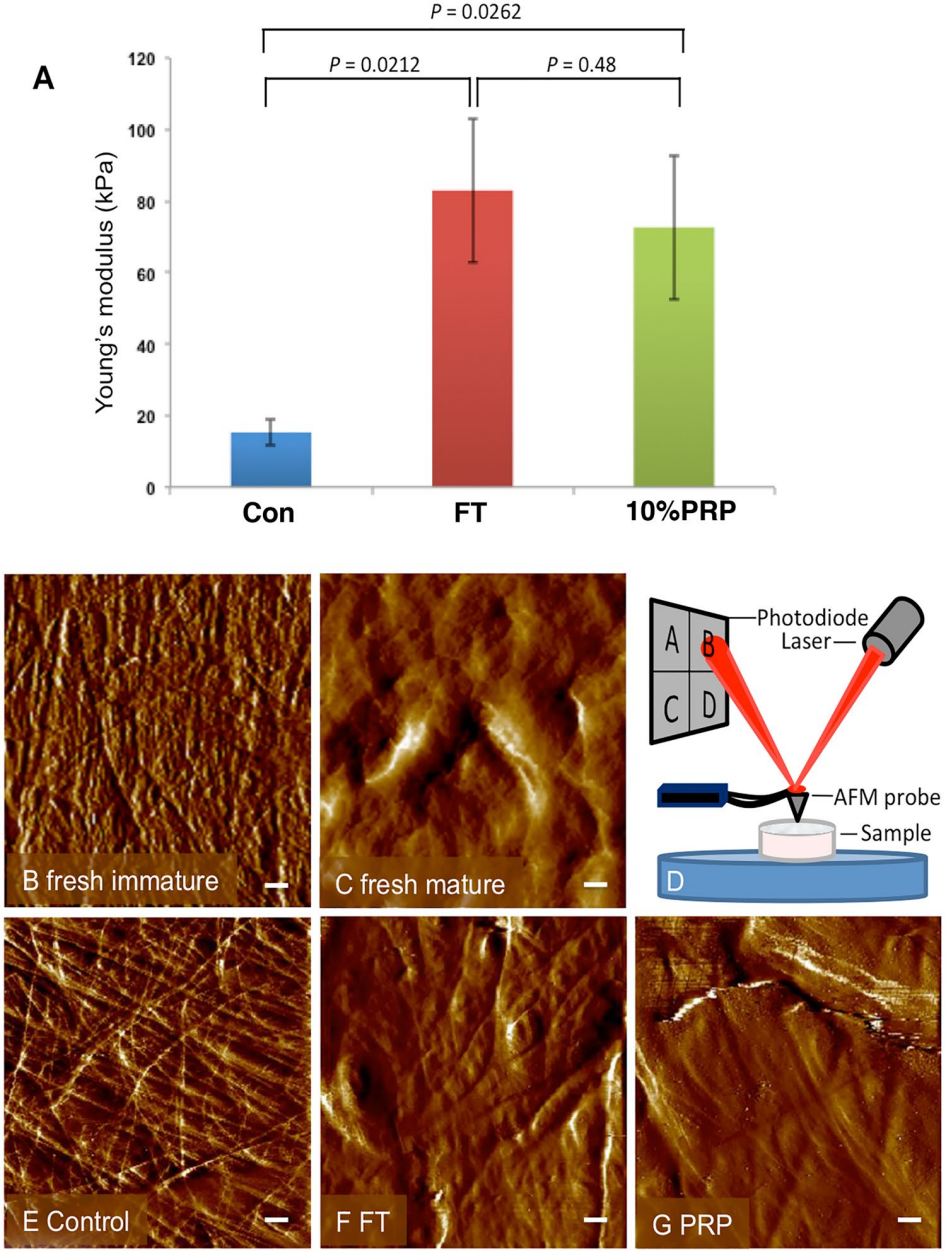
AFM analysis of the effect of PRP on cartilage stiffness and surface topology. Graph showing the change in stiffness (Young’s modulus, kPa) following treatment of cartilage explants for 21 days in culture (**A**). Surface topography of freshly isolated immature (**B**) and mature (**C**) cartilage explant as reference. Ten per cent PRP lysate (**G**) and growth factor (**F**) treated explants cultured for 21 days exhibited surface profiles that were similar to mature cartilage (**C**), whereas untreated explants (**E**) were closer in profile to freshly isolated immature cartilage (**B**), scale bar equals 0.5 μm. Diagram showing the experimental setup measuring cartilage biomechanical and topographical properties (**D**). The laser is deflected by the change in angle of the AFM probe and this deflection correlates with tissue biomechanical properties.

Culture of explants in PRP produces an increase in cartilage stiffness consistent with maturation. Incubation with 10% PRP caused a 4.7-fold increase in cartilage stiffness, measured by atomic force microscopy, (72.6 ± 20.15 kPa PRP v 15.33 ± 3.71 kPa control, *P* = *0.026*, n = 6), Fig. 3A. In comparison, growth factor treatment led to a comparable 5.4-fold increase in tissue stiffness (82.96 ± 20.12 kPa FGF2-TGFβ1, *P* = *0.021*, n = 6). Atomic force microscopy is also capable of obtaining topographical images of the apical surface of cartilage, Fig. 3D. We first scanned freshly isolated immature and mature cartilage bovine joints in order to generate reference images for qualitative analysis, Fig. 3B,C. The surface of immature cartilage explants cultured for three weeks in serum free medium were similar in appearance to freshly isolated explants of the same age, Fig. 3E. FGF2-TGFβ1 and 10% PRP treated immature cartilage explants were similar in surface smoothness to mature cartilage, Fig. 3F–G.

**Figure 4:**
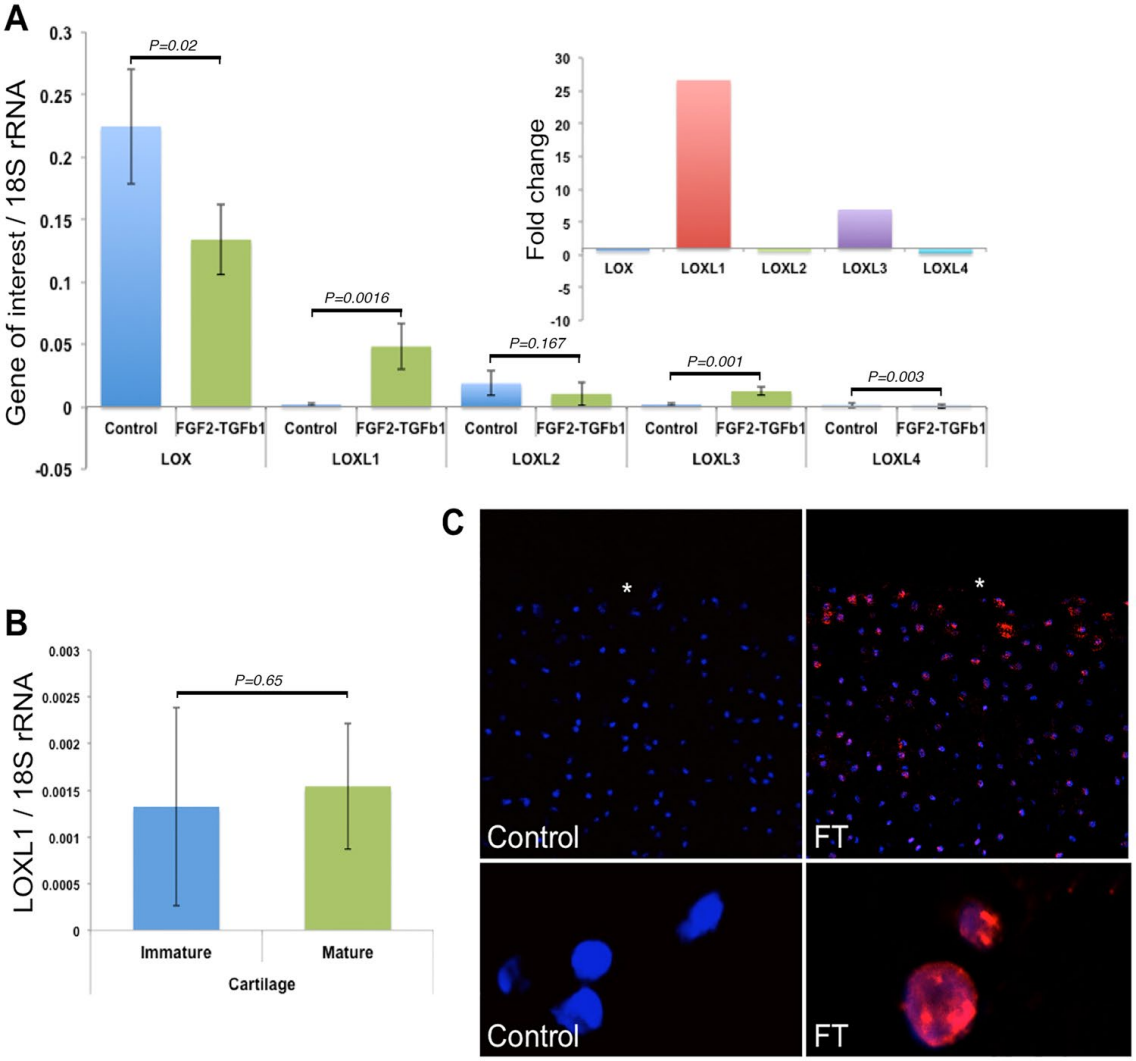
LOXL1 gene expression upregulation correlates with *in vitro* maturation of articular cartilage. Absolute gene expression values normalised to housekeeping gene 18S rRNA (in ng) for quantitative RT-PCR analysis of LOX isoform in control and *in vitro* growth factor-matured cartilage are shown (**A**). Relative values are also shown (*inset*). LOXL1 gene expression levels in freshly isolated immature and mature cartilage show no statistical difference (**B**). Rolling circle amplification *in situ* hybridisation shows LOXL1 gene transcription (red fluorescent labelling) is localised predominantly to the surface (white asterisk) of growth factor treated articular cartilage and is not detectable in untreated control cartilage where only nuclear counterstaining with DAPI is visible (**C**). The lower panel shows high magnification images of negative DAPI labelled nuclei and positively labelled (red) chondrocytes.

Collagen stiffness is a function of crosslinking, which is catalysed by the activity of a family of lysyl oxidases (LOX). Therefore, we measured the gene expression levels of all known LOX family isoforms in cartilage, Fig. 4A. During *in vitro* FGF2-TGFβ1 induced maturation, three LOX transcripts were downregulated, and LOXL1 and LOXL3 were significantly upregulated. In particular, LOXL1 gene expression was elevated 26.6-fold over control explants (*P* = *0.0016*. LOXL1 gene transcription was unchanged between freshly isolated immature and mature cartilage, indicating that transient elevation of LOXL1 gene expression is important in articular cartilage maturation, Fig. 4B. *In situ* hybridization using rolling circle amplification showed LOXL1 gene expression was predominately localised to the superficial zone of cartilage explants, Fig. 4C.

**Figure 5:**
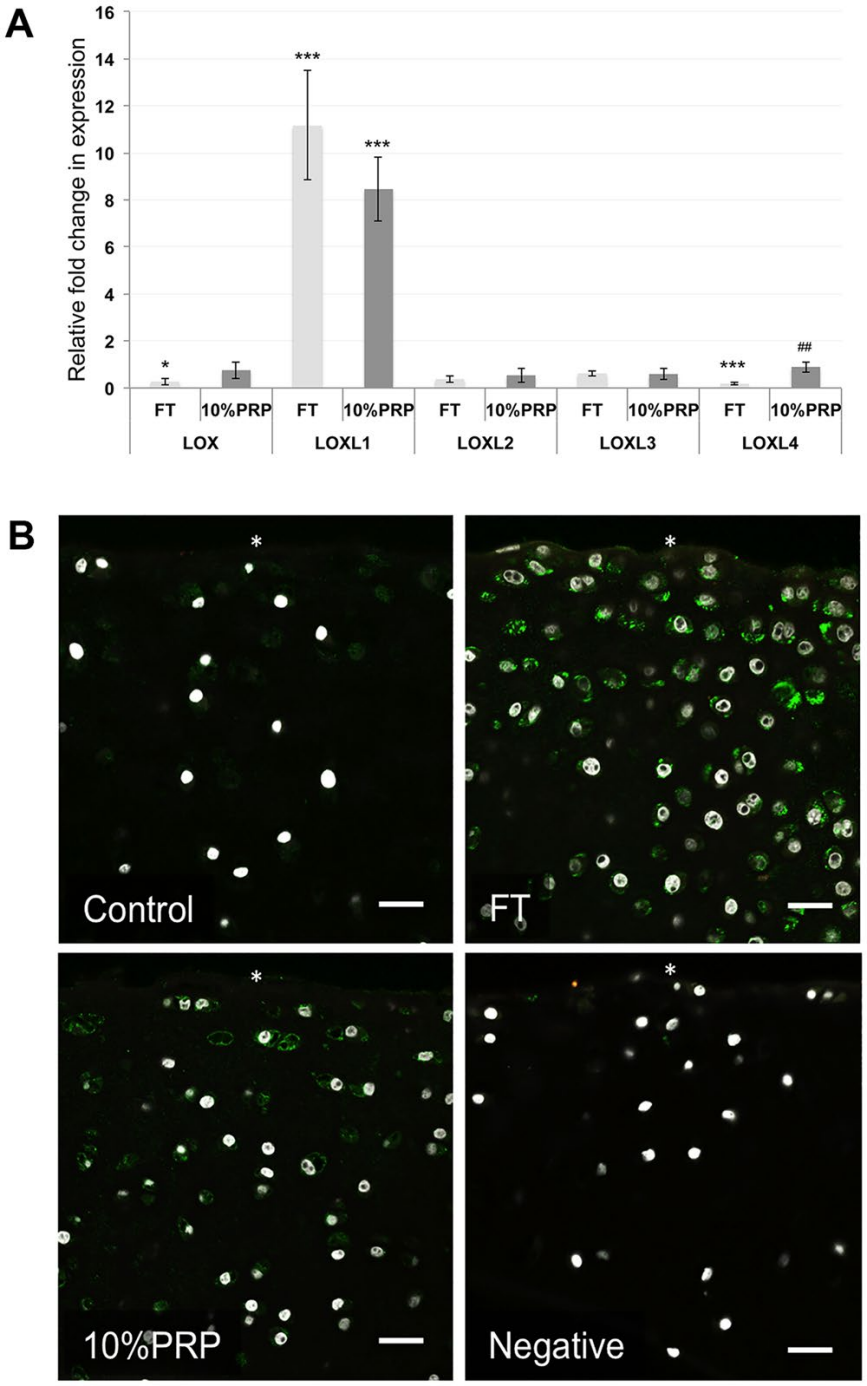
PRP upregulates LOXL1 gene expression and increases antibody labeling in the surface of immature cartilage explants. Graph showing the effect of 10% PRP and growth factors FGF2 and TGFβ1 (FT) treatment on the relative-fold gene expression of all known LOX isoforms compared to untreated explants. (**A**) The y-axis shows the relative fold change in gene expression calibrated to control samples. Significance values signified by * and *** equate to P < 0.05 and 0.001 versus control explants, and ## equates to P < 0.01 between treated groups. Confocal microscopy analysis of LOXL1 immunolabelling (green) and nuclei staining (white) in growth factor and 10% PRP treated cartilage (**B**). White asterisk denotes explant surface. The negative control, goat IgG, shows no positive labelling (**E**). Scale bar equals 20 μm.

PRP treatment activated LOXL1 gene expression in the surface of immature articular cartilage. Ten percent PRP induced LOXL1 gene expression 8.45-fold (*P* < *0.001*, n = 4) compared to an 11-fold increase in growth factor treated explants (*P* < *0.001*), Using these sample sets, expression levels were similar for all isoforms except LOXL4, which showed a 4-fold decrease compared to PRP treated explants. However, in the latter case there was no significant difference in gene expression of LOXL4 between explants cultured in control or PRP-containing medium. Figure 5A. LOXL1 protein immunolabelling was localised pericellularly and predominately in chondrocytes in the surface zone of growth factor and PRP treated explants, Fig. 5B–E.

**Figure 6:**
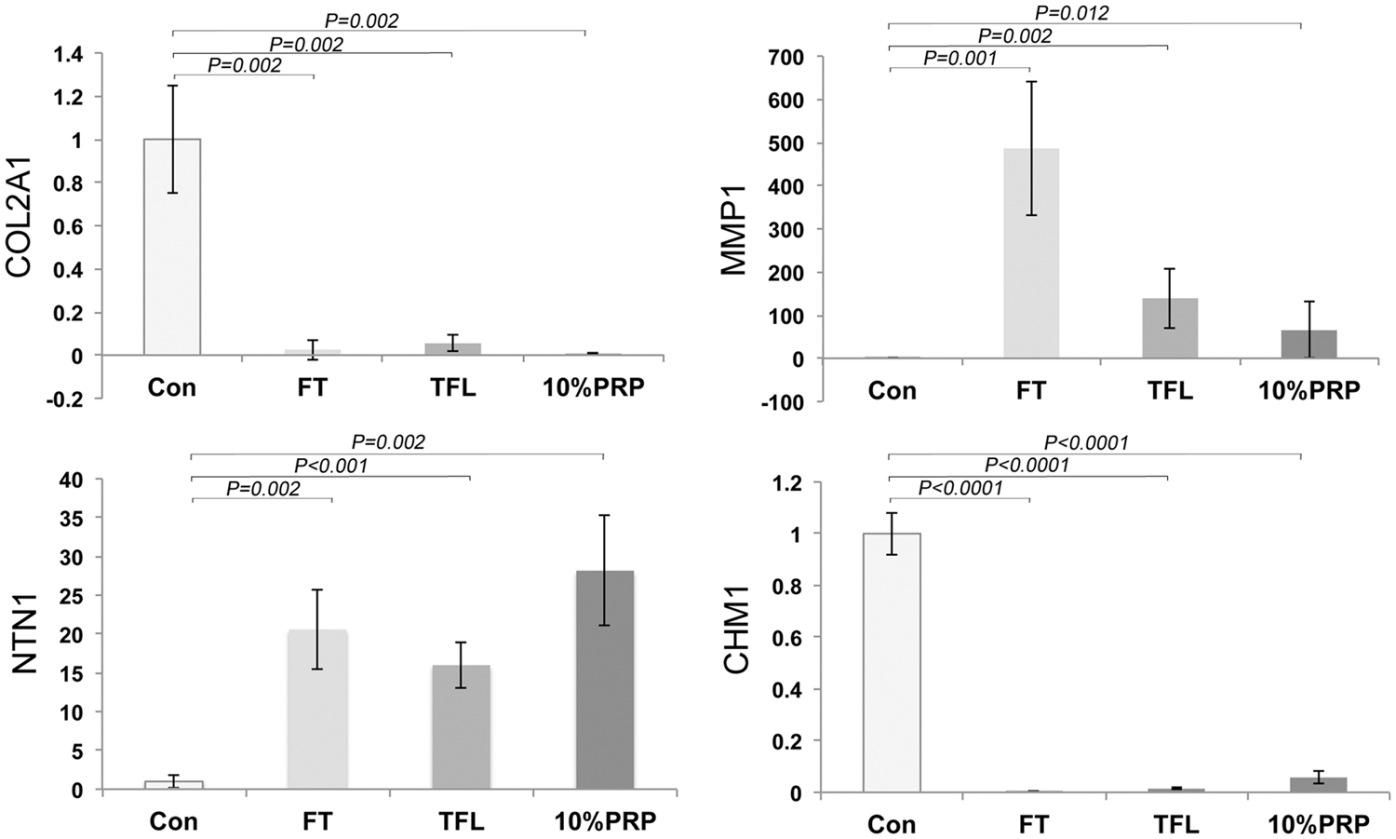
Growth factor concentrations of TGFβ1 and FGF2 comparable to those in PRP elicit similar gene expression profiles in cultured explants. Explants were cultured in medium containing 100 ng ml^−1^ FGF2 and 10 ng ml^−1^ TGFβ1 (FT), 10 ng ml^−1^ TGFβ1 and 17.5 pg ml^−1^ FGF2 (TFL), or, 10% PRP. The y-axis shows the relative fold change in gene expression calibrated to control samples.

Using elisa assay, the concentrations of FGF2 and TGFβ1 in PRP preparations were 176 ± 24 pg ml^−1^ and 169 ± 8 ng ml^−1^ (n = 5). Whilst the concentrations of TGFβ1 are comparable to those used *in vitro* to initiate maturation, the levels of FGF2 in PRP are approximately 6000-fold lower and in line with previously published values^28^. We therefore repeated RT-qPCR gene expression analysis of cartilage explants cultured using growth factor concentration comparable to that found in PRP, 10 ng ml^−1^ TGFβ1 and 17.5 pg ml^−1^ FGF2, Fig. 6. Our data shows that using the reduced FGF2 concentration elicited a similar gene expression profile as explants cultured in 10%PRP and standard growth factor concentrations used to induce *in vitro* maturation. The only difference between explants cultured with lower concentrations of FGF2 and those cultured with 10%PRP was where netrin-1 gene expression levels were 2-fold higher (*P* = *0.013*, n = 4) when cultured with PRP.

## Discussion

Repair of injured and diseased articular cartilage is a formidable proposition for clinicians and biologists alike due to its poor intrinsic healing capacity^15^. PRP has shown promise in reducing joint pain and increasing physical function^1^ especially in younger patients, but, without greater understanding of its mode of action, its function as a relatively simple therapeutic option is limited. Our discovery that PRP is able to induce salient features of articular cartilage maturation may provide a new rationale for its systematic use in joint repair.

Articular cartilage maturation is the fundamental developmental process that leads to the generation of adult tissue from an immature, isotropically organised cartilage template. Maturation differs from growth, the geometric increase in size of a tissue, in that continual remodeling of cartilage through a process of synchronised cellular proliferation and tissue resorption leads to the formation of an anisotropic, stiff and durable biological bearing surface^12, 14^. The importance of maturation in the context of repair relates to observations that show injured or diseased tissues adopting the phenotypic characteristics of immature cartilage. In surgical repair of osteochondral lesions using autologous chondrocyte implantation, transplanted chondrocytes do not recapitulate the pseudo-stratified structure of normal adult articular cartilage but mimic the isotropic organisation and gene expression profile of immature cartilage^23^. Also, it is well known that osteoarthritic articular cartilage undergoes a gradual dissolution of anisotropic organisation along with re-expression of proteins and epitopes usually found in embryonic and fetal cartilages^22^. Hunziker has argued that without maturational remodeling of cartilage, repair tissue is biomechanically compromised compared to surrounding cartilage and susceptible to further degenerative changes^11^. Therefore, stimulation of maturation is a key reparative process analogous to scar remodeling in wounded skin that over time regenerates near normal anatomy and full functionality.

*In vivo* maturation of articular cartilage in larger mammals take many months or years and was thought to be a difficult process to mimic *in vitro*. However, studies have shown that FGF2 and TGFβ1 induce precocious post-natal maturation-like changes in immature bovine articular cartilage^17^. *In vitro* growth factor-induced maturation in cultured explants stimulates synchronous proliferation of superficial zone chondrocytes and epiphyseal cartilage resorption, a decrease in collagen type II gene expression, an increase in the ratio of trivalent to divalent collagen crosslinks and increased tissue stiffness^17, 18^. The fact that maturation-dependent changes can be sensitively monitored *in vitro* make explant culture an ideal model system to test if factors, such as PRP, are capable of inducing this critical aspect of cartilage development and repair.

Our data shows PRP lysate is comparable to FGF2 and TGFβ1 in its ability to induce specific aspects of the maturational program. PRP treatment increased the cell density of surface chondrocytes in explants and this observation was supported by a proportionate increase in PCNA gene expression. Both PRP and FGF2-TGFβ1 induce proliferation of surface chondrocytes^17^, where the cells responding to maturational cues appear to be chondroprogenitors^14, 17, 29^ which display the cell surface characteristics and plasticity of mesenchymal stem cells (MSC)^30–32^. Studies show MSCs increase their proliferation rate in the presence of platelet lysate due to the presence of growth factors FGF2, TGFβ1, PDGFbb and IGF1^33, 34^. Similarly, studies have shown chondroprogenitors and dedifferentiated chondrocytes increase their proliferation by 3.7-fold when stimulated with purified FGF2, TGFβ1 and PDGFbb in monolayer^35^ and 1.5-fold in the presence of platelet-rich lysate^36^. Further support for the expansion of chondroprogenitors in treated cartilage comes from upregulated expression of integrin alpha-3 (ITGA3; also known as CD49c), a heterodimeric receptor with integrin β1 for fibronectin and a cell surface marker for the stage-specific transition of bone marrow-derived MSCs to a chondroprogenitor phenotype^37^. Dowthwaite *et al*. showed that articular chondroprogenitors preferentially bind to fibronectin, and used this property to enrich for colony forming cells^31^.

A key characteristic of maturing cartilage is an increase in tissue stiffness; both FGF2-TGFβ1 and PRP treatment of explants led to a 5-fold increase in their Young’s modulus compared to an untreated group. Studies have also shown that cartilage surface roughness decreases as cartilage matures and becomes stiffer^18^, and this was evident in FGF2-TGFβ1 and PRP treated cartilages that had qualitatively smoother surface profiles when compared to native and untreated immature explants. Increased cartilage stiffness is related to changes in collagen crosslinking^38^, and there is a direct correlation between an increase in pyridinoline crosslink concentration and the tensile strength of cartilage spanning from fetal to adult tissue^39^. Lysyl oxidases catalyse the first stage of the formation of pyridinoline crosslinks through oxidative deamination of lysine and hydroxylysine residues in collagen fibrils to form highly reactive allysine aldehydes. Deaminated amino acids spontaneously react with other allysine or lysine/hydroxylysine residues to form intra- and inter-fibrillar covalent crosslinks. We discovered that of the five LOX isoforms, LOXL1 gene was significantly upregulated in absolute terms during FGF2-TGFβ1-induced maturation and its transcripts were mainly localised in superficial zone chondrocytes. Moreover, LOXL1 expression appears to be transiently required for the maturational program as its expression is unchanged between immature and mature articular cartilage. Gene expression of LOXL1 was similarly induced by PRP treatment and protein expression confirmed by increased antibody labeling in surface zone chondrocytes. We saw variability in LOXL3 transcript levels between sample groups and this may be due to either variation in explant depth upon extraction or in ages of donor animals. However, LOXL1 transcript levels, which are localised to the articular surface, were consistently high when treated with growth factors or PRP. In summary, data shows LOXL1 presence is coincident with increased cartilage surface stiffness, and whilst the details of LOXL1 activity remains to be elucidated, it represents a novel transcriptional biomarker of maturation and also a potential target to activate collagen crosslinking specific for developmental repair mechanisms.

The concentrations of FGF2 to TGFβ1 used to initiate *in vitro* maturation were derived from previously published work that determined the minimal concentrations required to elicit maximal *in vitro* biological responses in intact explanted immature bovine cartilage^40, 41^. An analysis of published growth factor concentrations in PRP show that whilst TGFβ1 levels are broadly comparable to those used to induce *in vitro* maturation the published concentrations of FGF2 are up to 6,000 times lower^33^. Treatment of explants with concentrations of FGF2 comparable to those found in PRP elicited similar gene expression profiles for COL2, MMP1, NTN1 and CHM1. Studies have shown that concentrations of FGF2 released by human OA cartilage samples (28 pg FGF2 per 100 mg cartilage in 42 hours) are of a similar level as to those found in PRP preparations, and they correlate with MMP1 activity^42^. Vincent *et al*.^43^ have also shown that endogenous FGF2 released after cutting cartilage has profound effects on the tissue including strong induction of MMP1 and TIMP1 indicating its pivotal role in tissue remodeling^43^. Therefore, picogram concentrations of FGF2 in combination with TGFβ1 in PRP appear to be sufficient to induce some elements of the maturational program in young cartilage.

Our current understanding of the pathology of osteoarthritis suggests chondrocytes undergo progressive changes in phenotype extending from adoption of immature-like^44, 45^ and epiphyseal-like cell fates to reductions in growth factor responsiveness and senescence^46^. It is not surprising therefore to note that the most successful use of PRP for joint repair has been in younger patients, whose disease is in its earliest stages and where there is sufficient residual hyaline cartilage within which chondrocytes are still responsive to growth factor stimulation^22, 47^. A priori, our data proposes intra-articular injection of PRP may induce post-natal maturation of residual immature chondrocytes in injured or diseased cartilage to produce stiffer and more functional tissue^11, 17^. From the perspective of surgical cartilage repair PRP has been shown to induce chondrocyte proliferation, filling of the defect and differentiation^48–50^. In the latter studies PRP was loaded into acellular scaffolds which were then implanted into cartilage defects. PRP did not induce articular cartilage maturation in rabbits or humans as assessed using CHM1 gene expression or MRI T2 mapping^48, 50^. The latter studies did not test whether subsequent PRP application to the repaired cartilage could induce maturation within implanted scaffolds.

In conclusion, the major challenges in using PRP for cartilage repair have been the lack of knowledge regarding its mode of action and lack of standardisation of PRP preparations leading to inconsistent clinical results; current use is based on empirical evidence. This study has produced data consistent with the ability of PRP to induce articular cartilage maturation, a key developmental process that results in rapid growth and remodeling of immature cartilage to produce adult-like tissue. The maturation-inducing activity of PRP is context-dependent requiring the presence of an immature-like cartilage phenotype which is known to be re-expressed in early phases of tissue injury, disease and repair^21, 23, 44^. These data therefore provide the basis for a new biological rationale for evaluating the systematic use of PRP to treat injured or diseased cartilage either alone, or, as an adjuvant therapy to complement chondrocyte transplantation treatments, in order to accelerate remodeling of repair tissue to form stiffer and more durable cartilage.

## Material and Methods

### Preparation of bovine articular cartilage explants and culture

Cartilage explants were excised under sterile conditions from the lateral aspect of the medial condyle of the metacarpophalangeal joints of immature male bovine steers (obtained on day of slaughter from a local abattoir) using 6 mm diameter biopsy punches (Stiefel). All methods were carried out in accordance with relevant guidelines and institutional regulations. Explants in 24 well tissue culture dishes were incubated in serum-free medium consisting of high-glucose Dulbecco’s modified Eagle’s medium (DMEM), supplemented with insulin-transferrin-selenium (ITS, Invitrogen), 10 mM Hepes (pH 7·5), 50 μg mL^−1^ of gentamicin, and 50 μg mL^−1^ of sodium ascorbate at 37 °C in 5% CO_2_. Explants were cultured for 14 days for RNA analysis or 21 days for immunochemical and histological analyses with medium changed every third day.

### Growth factors & PRP treatments

FGF2, 100 ng mL^−1^ and TGFβ1, 10 ng mL^−1^ (Peprotech), were used in serum-free culture medium to induce maturation in immature cartilage explants. Expired human apheresis platelets in plasma serum were obtained from the Welsh Blood Service (Pontyclun, Wales) and resuspended at a concentration of 1 × 10^9^ platelets mL^−1^. The platelets underwent a single freeze-thaw cycle to form a platelet lysate containing released growth factors. The optimum concentration of lysed apheresis PRP used for this study was selected by quantitative RT-PCR to be the concentration at which the maximum response was achieved. For atomic force microscopy, the lysed platelets were placed into a transwell insert (6 mm diameter, 0.4 m pore size; Millipore) to prevent the weak gel produced by the lysate from interfering with cartilage surface topography. The concentration of FGF2 and TGFβ1 in apheresis PRP preparation were quantified using Human Quantikine elisa kits (R&D Systems, UK). Measurements of growth factor concentration fell within the linear range and sensitivity of the assays.

### Bromodeoxyuridine (BrdU) incorporation assays

Forty-eight hours prior to the end of explant culture, BrdU, 10 μM, was added to the culture medium. Explants were fixed in 10% neutral-buffered formalin (NBF) then embedded in wax and sectioned at 5 μm thickness. Hydrated sections were placed for 30 minutes in 1N HCl, then in 0.1 M borate buffer, pH 8.0. Following blocking with goat serum they were incubated with 1:5 dilution anti-BrdU antibody G3G4 (Developmental Studies Hybridoma Bank, University of Iowa) in phosphate buffered salts containing 0·1% (v/v) Tween-20 (PBST). Goat anti-mouse IgG alexa fluor 594 conjugated antibody (Invitrogen) at 5 μg mL^−1^ was used to localise primary antibody.

### Quantitative reverse transcription and polymerase chain reaction (qRT-PCR)

Primers for 18S rRNA, lysyl oxidase (LOX) and its isoforms L1-4, collagen types II/X (COL), matrix metalloproteinase-1 (MMP1), netrin-1 (NTN1), integrin alpha-3 (ITGA3), deiodinase-2 (DIO2) and chondromodulin-1 (CHM1) were designed using the National Center of Biotechnology Information online resource. A standard curve made using each cloned PCR product was used to determine absolute values for amplified products normalised to 18S rRNA. Complimentary DNA (cDNA) was prepared from RNA isolated from frozen cartilage explants homogenized using a Mikro-dismembrator U in the presence of TRI Reagent (Sigma) as previously described, Total RNA was further purified using RNeasy minicolumns with a DNAse cleanup step (Qiagen). cDNA was prepared using 500 ng total RNA per reaction. Samples for qRT-PCR reaction were run in duplicate using GoTag qPCR Master Mix (A6002; Promega), 5 ng of cDNA, and 0.3 mM forward and reverse primers. The thermal cycling program used was: 95 °C for 10 minutes for 1 cycle, then 40 cycles of 95 °C for 30 seconds, 55 °C for 60 seconds, and 72 °C for 30 seconds. Bovine specific primer sequences are shown in Supplemental Data 1. PCR products were cloned and sequenced to confirm identities.

### Immunofluoresence detection of lysyl oxidase L1

Wax embedded sections were deparaffinised, hydrated and pre-treated with a cocktail of 1 mg mL^−1^ bovine testicular hyaluronidase (H3506; Sigma) and 0.1 U mL^−1^ chondroitinase ABC (C2905; Sigma) in PBST for one hour at 37 °C to remove glycosaminoglycans. Blocking serum 10% (v/v) donkey serum was applied for one hour, followed by primary antibody 1:50 dilution goat anti-LOXL1 (N-20, sc-48720; Santa Cruz Biotech). Donkey anti-goat IgG alexa fluor 594 conjugated antibody at 5 μg mL^−1^ were used to visualise primary antibodies. Negative controls without primary antibody and goat IgG were used to confirm antibody binding specificity.

### Rolling circle amplification (RCA) *in situ* hybridization detection of LOXL1 transcripts

Ten micron cryosections of cartilage explants were processed for RCA *in situ* exactly according to Larrson *et al*.^51^. Briefly sections were fixed, incubated with 0.01% pepsin and then air dried. Sections were then subjected to reverse transcription using RevertAid H minus M-MuLV reverse transcriptase, RNAse inhibitor (Fermentas) and a Locked Nucleic Acid modified cDNA primer (Supplemental Data 1). A 57-nucleotide ‘padlock’ probe was hybridized to the cDNA and subsequently ligated to form circular templates for transcription using Φ29 DNA polymerase. Finally, hybridization of a fluorescently labeled oligonucleotide 488-M13R to a complementary sequence in the padlock probe allowed detection by fluorescence microscopy, BX61 (Olympus).

### Atomic force microscopy (AFM) analysis

Nanoindentation experiments were performed using a Nanowizard II (JPK, Germany) instrument. Imaging experiments were performed using the quantitative nanomechanical mapping (QNM) capabilities of a Bioscope Catalyst (Bruker, USA) instrument. High aspect ratio etched silicon probes, dNP-10 and MLCT (Bruker) both of radius 20 nm and spring constants of 0.32 Nm^−1^ and 0.03 Nm^−1^ with resonant frequencies of 40–75 kHz and 26–50 kHz, respectively. Bovine explants (6 mm diameter) were immobilised onto the surface of glass-bottomed Petri dishes in DMEM. The cantilever approach and retraction velocity was constant, set at 3.0 μm s^−1^. The Poisson ratio was assumed to equal 0·5. Nanoindentation force experiments were conducted capturing 64 indentation curves in each scan area (5 × 5 μm). These data represented the basis for measurements of a sample’s Young’s modulus (E) using Hertzian mechanics.

### Statistical analysis

Data were presented as mean ± standard deviation (SD). All data sets were checked for normal distribution using the Shapiro-Wilk test and for homogeneity of variances using Levene’s test prior to parametric analysis. A one-way analysis of variance (ANOVA) test was used to analyse differences between multiple groups. Non-parametric Kruksal-Wallis and Mann-Whitney *U* tests were used where data was either not normally distributed or variances not equal. Data sets were analysed using PASW Statistics 20, release version 20.0.0 (SPSS). Statistical significance was assigned as *P* < 0.05.

## Electronic supplementary material

Supplementary Dataset 1
