# Supplementary material for: Platelet-rich plasma induces post-natal maturation of immature articular cartilage and correlates with LOXL1 activation
Source: Sci Rep. 2017 Jun 16;7:3699. doi: 10.1038/s41598-017-02297-9 (PMC5473810; doi:10.1038/s41598-017-02297-9)

**Supplemental data**: Platelet-rich plasma induces post-natal maturation of immature articular cartilage and correlates with LOXL1 activation

^1^Yadan Zhang PhD, ^1,2^Ben J. Morgan PhD, ^1^Rachel Smith PhD, ^4^Christopher Fellows PhD, ^3^Catherine Thornton PhD, ^4^Martyn Snow MBBS, MSc, ^2^Lewis Francis PhD, ^1^Ilyas M. Khan PhD*.

^1^Centre of NanoHealth, ^2^Reproductive Biology Group, ^3^Human Immunology Group, Swansea University Medical School, Swansea University, Singleton Park, Swansea, SA2 8PP, United Kingdom. ^4^School of Veterinary Medicine, Surrey University, Guildford, Surrey, GU2 7AL, United Kingdom. ^4^Royal Orthopaedic Hospital, Northfield, Birmingham, B31 2AP, United Kingdom

| **Supplemental data 1.** | |  |
| --- | --- | --- |
|  |  |  |
| Name | Primer sequence | size nt |
|  |  |  |
| 18SrRNA_F | CACTGGAGGCCTACACGCCG | 20 |
| 18SrRNA_B | AGGCAATTTTCCGCCGCCCA | 20 |
| bLOXL_F | GCGACGACCCGTACAACCCC | 20 |
| bLOXL_B | TGCTGAGCTGGCCAAGCAGTT | 21 |
| bLOXL1_F | GCGTTTCCCCCAGCGTGTGA | 20 |
| bLOXL1_B | GCTGTGGTAGTGCTGGTGGCA | 21 |
| bLOXL2_F | TCGCCTGCTCAGAAACCGCC | 20 |
| bLOXL2_B | CATGGCGCCCGTTCTTGGGT | 20 |
| bLOXL3_F | CGGCTGGCTGGTTTCCCCAG | 20 |
| bLOXL3_B | GCCAGATGCGGCCTGTTCCA | 20 |
| bLOXL4_F | TCGACAGCCACCACTACAGGAAAGT | 25 |
| bLOXL4_B | TGCCCAGGCAGTTGACACGG | 20 |
| bRPS18_F | CACTGGAGGCCTACACGCCG | 20 |
| bRPS18_B | AGGCAATTTTCCGCCGCCCA | 20 |
| bCOL2A1_F | CTGGATGCCATGAAGGTTTT | 20 |
| bCOL2A1_B | GCTCCACCAGTTCTTCTTGG | 20 |
| bITGA3_F | AGCCATCCATACTGGACCCT | 20 |
| bITGA3_B | ATGTTCCGCCTGTAGTTGGG | 20 |
| bDIO2_F | GCCCCCAATTCCAGCGTGGT | 20 |
| bDIO2_B | AGGTCAAGTGGCCGAGCCGA | 20 |
| bNTN1_F | TCACGGATCTCAACAACCCG | 20 |
| bNTN1_B | GTAGATGGCCATGGACTCGC | 20 |
| bCOLX_F | CCCATGCTTGGGTAGGTCTG | 20 |
| bCOLX_B | CCATACCTGGTCGTTCTCGG | 20 |
| bCHM1_F | GCTACATCAAAGCGCAGGTG | 20 |
| bCHM1_B | GATCTCCAGCCACCCAGATG | 20 |
| bPCNA_F | GTCAGGAGTCAACCAAGA | 18 |
| bPCNA_B | GGATACAGTGAGTTCTACCA | 20 |
| *cLOXL1 | **GAAGGAGAAGCACGCACGGGGG**AATAA**CGCCAGGGTTTT** |  |
|  | **CCCAGTCACGA**CAATAA**GTCCTGTCCTCGAGGCCGTG** | 76 |
| **cDNALOXL1 | G*GG*CC*AG*AG*CCATGGTGG | 18 |
| FITCDP | CGCCAGGGTTTTCCCAGTCACGA | 23 |

* The padlock probe used in this study was designed using the Bos *taurus* reference mRNA sequence for LOXL1 deposited in Genbank (NM_174383). The oligonucleotide is composed of a 20nt 3' sequence hybridising to LOXL1 cDNA a 5nt stuffer sequence (AATAA) a 22nt detection probe, a stuffer sequence followed by a 5' 21nt sequence that hybridises contiguous with the 3' of the oligonucleotide. The hybridised padlock sequence is circularised using DNA ligase, therefore the oligonucleotide must contain a phosphorylated 5’ nucleotide to allow ligation. In addition it is recommended that the oligonucleotide be PAGE-purified to increase the efficiency of circular DNA formation (see Supplemental Data, Larsson *et al*, 2010). A Texas red labelled detection oligonucleotide (TR-DP) was used to detect rolling circle amplified DNA using florescence microscopy.

** the cDNA priming oligonucleotide was synthesised with 5 Locked Nucleic Acid (LNA) modifications (*N) as recommended by Larsson et al (2010)

Larsson C, Grundberg I, Soderberg O, Nilsson M. *In situ* detection and genotyping of individual mRNA molecules. *Nature methods*. **2010**; 7(5): 395-7.


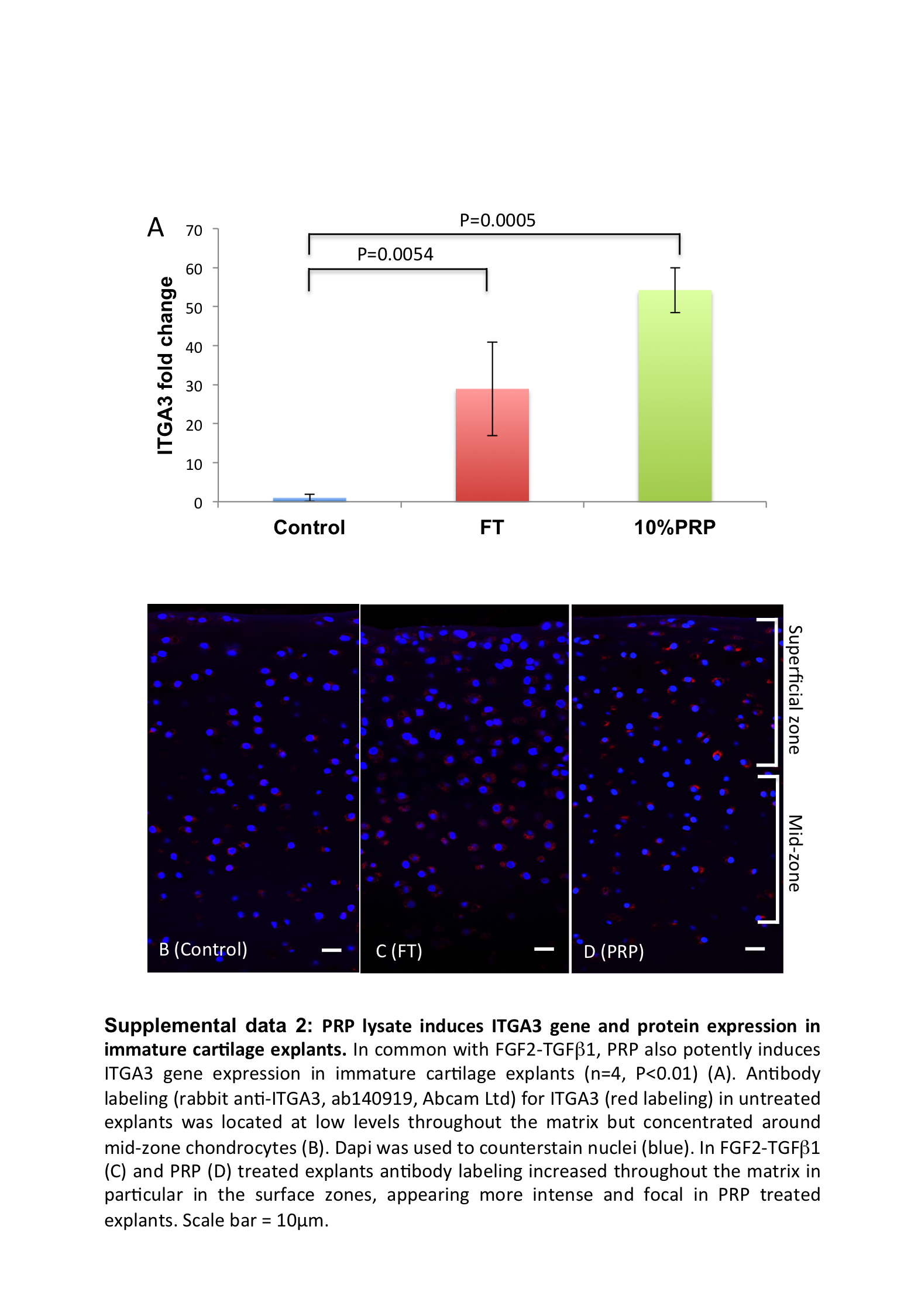

Supplement: Supplementary file 1 — Supplementary Dataset 1 [file 41598_2017_2297_MOESM1_ESM.doc]
